# Percutaneous Extraction of Transvenous Permanent Pacemaker/Defibrillator Leads

**DOI:** 10.1155/2014/949785

**Published:** 2014-05-26

**Authors:** Stylianos Paraskevaidis, Dimitrios Konstantinou, Vassilios Vassilikos, Efstratios Theofilogiannakos, Lilian Mantziari, Athanasia Megarisiotou, Ioannis Galitsianos, Charalambos Karvounis

**Affiliations:** ^1^First Department of Cardiology, AHEPA University Hospital, Aristotle University Medical School, 1 St. Kyriakidi Street, 54636 Thessaloniki, Greece; ^2^Third Department of Cardiology, Hippokrateion University Hospital, Aristotle University Medical School, Thessaloniki, Greece; ^3^Department of Cardiology, Electrophysiology Unit, Royal Brompton Hospital, London, UK

## Abstract

*Background*. Widespread use of cardiovascular implantable electronic devices has inevitably increased the need for lead revision/replacement. We report our experience in percutaneous extraction of transvenous permanent pacemaker/defibrillator leads. *Methods.* Thirty-six patients admitted to our centre from September 2005 through October 2012 for percutaneous lead extraction were included. Lead removal was attempted using Spectranetics traction-type system (Spectranetics Corp., Colorado, CO, USA) and VascoExtor countertraction-type system (Vascomed GmbH, Weil am Rhein, Germany). *Results.* Lead extraction was attempted in 59 leads from 36 patients (27 men), mean ± SD age 61 ± 5 years, with permanent pacemaker (*n* = 25), defibrillator (*n* = 8), or cardiac resynchronisation therapy (*n* = 3) with a mean ± SD implant duration of 50 ± 23 months. The indications for lead removal included pocket infection (*n* = 23), endocarditis (*n* = 2), and ventricular (*n* = 10) and atrial lead dysfunction (*n* = 1). Traction device was used for 33 leads and countertraction device for 26 leads. Mean ± SD fluoroscopy time was 4 ± 2 minutes/lead for leads implanted <48 months (*n* = 38) and 7 ± 3 minutes/lead for leads implanted >48 months (*n* = 21), *P* = 0.03. Complete procedural success rate was 91.7% and clinical procedural success rate was 100%, while lead procedural success rate was 95%. *Conclusions.* In conclusion, percutaneous extraction of transvenous permanent pacemaker/defibrillator leads using dedicated removal tools is both feasible and safe.

## 1. Introduction

Widespread use of cardiovascular implantable electronic devices (CIED) has inevitably resulted in higher necessity for removal of infected or dysfunctional leads. Extraction of these leads has been effected using a wide variety of percutaneous techniques including simple traction, continuous traction, polymer or metal sheath system plus locking stylets, and more recently devices using laser or radiofrequency energy, to assist in sheath insertion. Despite high reported rates of successful explantation [1–3], the procedure is still costly and time consuming while highly trained and experienced operators need to be involved. Percutaneous removal of CIED leads can be a challenging procedure, accompanied by serious complications [1, 3], especially when long standing electrodes are involved with scar tissue encountered along the vein and within the heart. We report our experience in percutaneous extraction of transvenous permanent pacemaker/defibrillator leads using traction or countertraction removal systems. Of note, no laser sheaths were employed in any of the extractions attempted in this study.

## 2. Methods

A consecutive series of 36 patients who were admitted to our centre from September 2005 through October 2012 for percutaneous lead extraction were retrospectively identified. The clinical and pacing data were retrieved from patient records and from our electrophysiology laboratory database.

Indications for extraction were infection and mechanical lead failure/malfunction. Infection was defined as either pocket infection with local signs of erythema, pain, or purulent discharge or infective endocarditis in the presence of permanent transvenous leads. In particular, infective endocarditis was diagnosed in each case where persistent bacteraemia or sepsis was noted or noninvasive imaging revealed vegetation on the leads or on the valves in the presence of a CIED. Lead malfunction was considered when clinically significant changes in lead sensing, pacing, or threshold parameters were observed during periodic device follow-up.

Extraction was performed in the electrophysiology laboratory via the implant vein, under local anaesthesia and mild sedation with midazolam where needed. In pacemaker dependent patients, a temporary pacing wire was inserted via the right femoral vein in advance. Subsequently, the pocket was exposed and the generator was retrieved and disconnected from the lead(s). In case of active fixation leads the mechanism was unscrewed if possible. As soon as the lead was freed from the subcutaneous tissue, a conventional stylet was advanced under fluoroscopic guidance up to the tip of the lead. This stylet was then exchanged with a locking stylet and lead extraction was attempted.

**Figure 1:**
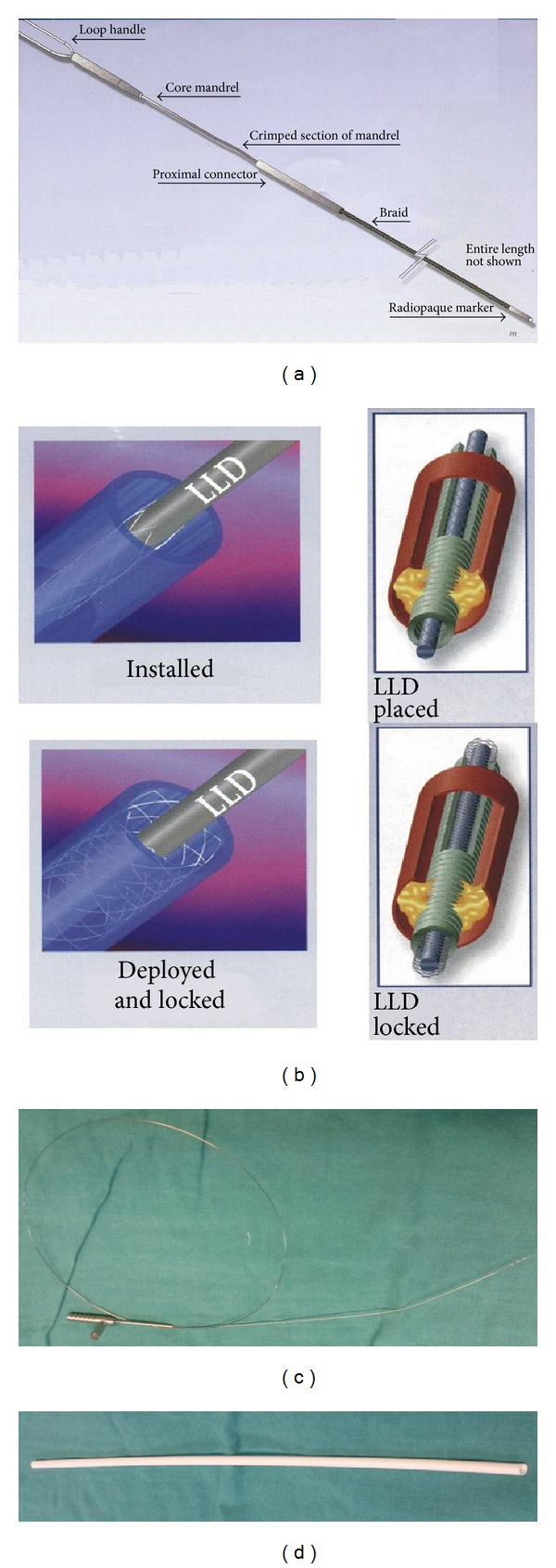
(a) Spectranetics system for percutaneous lead extraction. Traction locking device (Spectranetics Corp., Colorado, USA); (b) schematic representation of Spectranetics system: placement, expansion, and fixation of the device; (c) VascoExtor system for percutaneous lead extraction. Countertraction device (VascoExtor, Vascomed GmbH, Weil am Rhein, Germany): special locking stylet and (d) dilator sheath.

Two different dedicated lead removal tools were employed: Spectranetics traction-type system (Spectranetics Corp., Colorado, USA) and VascoExtor countertraction-type system (Vascomed GmbH, Weil am Rhein, Germany). The traction-type device consists of a special locking stylet which is advanced through the coil lumen of the lead and then is being locked in place at the lead tip (Figure 1(a)). After stent-like expansion inside lead lumen (extending from the myocardial wall up to the subclavian or cephalic vein), placing firm traction on the locking stylet can free the lead (Figure 1(b)). The countertraction device consists of a special locking stylet inserted in the lead lumen and locked in place at the lead tip (Figure 1(c)) and a set of telescoping dilator sheaths advanced over the lead under fluoroscopy (Figure 1(d)). After careful manipulation and traction on the stylet, while the sheath counters the traction and supports the myocardial or the vein wall, the scar tissue can be disrupted and the lead can be freed (Figure 1(d)). The VascoExtor countertraction-type system (Vascomed GmbH, Weil am Rhein, Germany) which was used in the present study was not a laser sheath.

Complete procedural success rate was defined as the number of cases where complete removal of all targeted leads was achieved divided by the number of procedures performed [4]. Clinical procedural success rate was calculated by dividing the number of cases where all targeted clinical goals of the procedure were met by the number of the procedures [4]. Hence, incomplete removal of all components of an intravascular lead was considered as clinical failure if the initial indication for lead extraction was infection. In case of lead malfunction, however, the same scenario would be documented as clinically successful procedure. Finally, lead clinical success rate was equal to the number of successfully explanted leads divided by the total number of leads where extraction was attempted [4].

## 3. Results

A total of 36 patients (27 men) were identified with a mean ± SD age of 60 ± 5 years (range 45–80 years). Among them, 25 (69.4%) had a pacemaker, 8 (22.2%) had an implantable cardioverter defibrillator (ICD), and 3 (8.4%) had a cardiac resynchronisation therapy-defibrillator (CRT-D) in situ. The indication for lead removal was pocket infection in 23 (63.9%) patients, endocarditis in 2 (5.5%) patients, ventricular lead dysfunction in 10 (27.8%) patients, and atrial lead dysfunction in 1 (2.8%) patient. Patients' demographics, device type, and indication for lead extraction are summarized in Table 1.

Lead extraction was attempted in 59 leads including 20 ventricular pacing leads, 21 atrial pacing leads, 5 VDD leads, 10 ventricular pacing-defibrillator leads, and three coronary sinus leads. The mean ± SD time elapsed since lead implantation was 50 ± 23 months (range 9–120 months). All except three atrial pacemaker leads were passively fixated. Traction-type system Spectranetics (Spectranetics Corp., Colorado, USA) was used in 33 leads (55.9%) and countertraction-type system VascoExtor (Vascomed GmbH, Weil am Rhein, Germany) in 26 leads (44.1%). Lead characteristics and extraction method used are outlined in Table 1.

The mean ± SD fluoroscopy time was 4 ± 2 minutes/lead for leads implanted <48 months (*n* = 38) and 7 ± 3 minutes/lead for leads implanted >48 months (*n* = 21), *P* = 0.03. The mean ± SD procedure time was 80 ± 15 minutes without taking into account the additional time needed for a new device implantation in ad hoc cases.

The procedure was prematurely terminated due to severe chest pain in one patient with a DDD pacemaker and ventricular lead dysfunction using the Spectranetics device. As a result of this, the ventricular lead was left in place and a new ventricular lead was implanted instead. Extraction failure was noted in 4 dysfunctional ventricular leads (2 pacing and 2 pacing-defibrillator leads) owing to firm adhesions to the subclavian vein, despite an initial successful lead detachment from the myocardial wall. Both ventricular pacing leads were eventually removed via femoral vein using a snare extractor (Vascomed GmbH, Weil am Rhein, Germany). During the extraction of a ventricular pacing lead, ventricular fibrillation was induced mandating external cardioversion. The actual timing of the VF coincided with the detachment of the ventricular pacing lead from the right ventricular wall. Regarding the 2 pacing-defibrillator leads, they were left inside. Of note, all four patients where extraction was failed had VVI devices in situ; therefore no atrial leads had to be extracted.

Complete procedural success rate was 91.7% and lead procedural success rate was 95%. However, clinical procedural success rate was considered to be 100% as incomplete lead removal noted in 3 cases involved noninfected leads. All 3 patients subsequently received a new functional lead.

A new ventricular electrode was implanted during the same procedure as the extraction, in 10 patients with ventricular lead dysfunction (8 pacing leads and 2 pacing-defibrillator leads) and a new atrial lead in 1 patient with atrial lead dysfunction. In patients with pocket infection/endocarditis a new device was implanted after resolution of the infection at the contralateral site. Among them, a new pacemaker was implanted in 17 patients (12 DDDR and 5 VVIR devices), a new DDDR ICD in 5 patients, and a new CRT-D in 3 patients. In 23 patients with pocket infection, a new device was implanted when the blood cultures obtained 24 hours following device removal remained negative for 72 hours. In 2 patients with device-related endocarditis, a more conservative approach was undertaken and the new permanent system was implanted after they have completed a 4-week course of iv antibiotics.

No major complications (infection or dysfunction) were observed in the newly implanted leads/devices during a mean follow-up of 12 months. Of note, an abrupt and prominent elevation in ventricular pacing threshold (5 Volt pulse amplitude at 1 ms pulse width) occurred in 3 (8.3%) patients, two days after implantation of a new DDDR pacing system (lead and generator) which persisted for 1 year. Both of the abovementioned leads were bipolar, passive fixation ventricular leads. This elevation was possibly due to initial oedema and late fibrosis in the area of extraction and reimplant despite the best of our efforts to avoid implanting the new leads in the same position as the extracted ones. Gradual resumption of the pacing threshold elevation was noted after 12 months.

## 4. Discussion

The need for extraction of infected or failing pacemaker/defibrillator leads becomes an increasingly encountered clinical problem, considering the constantly rising device implantations rates, the number of implanted leads per patient, and patient related factors including advanced age, frailty, and comorbidities. We reported a series of 36 patients where extraction of a total of 59 leads was attempted. Despite using basic extraction tools, that is, traction and countertraction techniques with no laser powered sheaths back-up, our overall success rates were considerably high.

According to Heart Rhythm Society latest expert consensus statement on lead extraction [4], class I indications include (1) infection, (2) thrombosis or venous stenosis, and (3) directly lead related issues involving functional or nonfunctional leads. Regarding infection, class I indications for complete device and lead removal include definite device-related endocarditis, pocket infection with abscess formation, skin erosion, or chronic purulent discharge (even if there is no conclusive evidence of involvement of the intravascular part of the lead), infective endocarditis affecting a cardiac valve without definite involvement of the lead, and occult gram-positive bacteraemia in patients with a CIED in situ (excluding cases of contamination). In case of venous thrombosis/stenosis, lead removal is indicated in patients with clinically significant thromboembolic complications associated with a lead or a lead fragment, bilateral subclavian vein occlusion, or superior vena cava occlusion precluding the placement of a needed lead, superior vena cava syndrome, or in case of ipsilateral subclavian vein occlusion precluding the insertion of a new lead when access via the contralateral site is not an option. Lead related class I indications for lead removal include life-threatening arrhythmias secondary to retained leads, interference between the abandoned lead and another functional lead and in case where a lead interferes with the treatment for malignancy such as radiation or reconstructive surgery.

In terms of advisory leads, relevant recommendations are provided by the lead performance policies and guidelines issued by Heart Rhythm Society [5]. An advisory lead should be revised or replaced if the risk associated with lead malfunction is likely to cause the patient significant harm or even death. This is particularly relevant to patients who are pacemaker dependent or have an ICD in situ for secondary prevention or have experienced the past appropriate ICD discharges. In patients who do not meet the above criteria and whose operative risk probably outweighs the anticipated benefits from lead revision/replacement, they should be managed conservatively with frequent monitoring. In all patients, reprogramming of the device should be contemplated if this can reduce the risk of possible lead malfunction to the patient. In our series, the indication for transvenous lead removal was erosion or pocket infection in 23 patients, endocarditis in 2 patients, and lead dysfunction in 11 patients.

Techniques for transvenous lead removal are considered: (a) simple traction, efficacious only during the first months after implantation, (b) traction using special devices or locking stylets, (c) countertraction, using a special locking stylet and a set of telescoping dilator sheaths [6], (d) laser sheaths which have significantly improved lead extraction efficacy [2], and (e) lead removal via inferior vena cava and femoral vein using special catheters (basket snare, pigtail, and Amplatz) to engage or entrap and remove the lead or lead fragments [7, 8]. These techniques are in use since late 1980s and early 1990s. In our case series, the traction-type system Spectranetics (Spectranetics Corp., Colorado, USA) was used in 33 leads and the countertraction-type system VascoExtor (Vascomed GmbH, Weil am Rhein, Germany) in 26 leads.

In an early multicentre study where a total of 3,540 leads were extracted from 2,338 patients, complete removal was achieved for 93% of leads, partial for 5%, while 2% were not removed [1]. In the PLEXES trial, the efficacy and safety of laser sheaths were tested against conventional lead extraction methods in 301 patients with 465 chronically implanted pacemaker leads [2]. Complete lead removal rate was significantly higher in the laser group and was reported to be up to 94%. More recently, the LEXICON study reported the outcomes of laser-assisted extraction of 2,405 leads in 1,449 consecutive patients [3]. Overall, procedural success rate associated with complete lead removal was 96.5% whereas the procedure was considered to be clinically successful in 97.7% of the cases.

In our cohort, complete procedural success rate was achieved in 91.7% of the cases and lead clinical success rate was 95%. Of note, clinical procedural success rate was equal to 100% suggesting that clinical goals associated with the indication for lead removal were achieved in all the participants. This high procedural success rate despite using relatively outdated extraction techniques may be attributed to certain patients' characteristics including older age and infection as the main indication for lead extraction. Byrd et al. [1] reported that incomplete/failed extraction was more likely to occur in younger patients as the extent and quality of the scar tissue formed around the leads are inversely related to age. In a study by Bracke et al. [9] it was reported that infection as an indication for lead extraction was a multivariate predictor of successful lead removal with simple traction. Nevertheless, had we used laser powered sheaths, a complete procedural success rate might be even higher approaching that reported in the literature [10].

In a large, single-centre study where 975 chronic endovascular leads were removed from 498 patients, leads implanted longer than 3.4 years and ICD leads were more likely to mandate the use of laser sheath [10]. In the LEXICON study, multivariate predictors of failure to achieve procedural success were leads implanted for ≥10 years and when extraction performed in a low-volume centre [3]. In our cohort, leads implanted >48 months were associated with significantly prolonged fluoroscopy time compared to leads implanted <48 months. However, lead age per se did not have a negative impact on lead extraction success rate. Overall failure to remove a lead via the implant vein was 6.8% and this was equally distributed between ventricular pacing (*n* = 2) and defibrillator leads (*n* = 2).

Percutaneous transvenous lead removal procedures can potentially be complicated by major cardiovascular adverse events including tricuspid valve injury or rupture, pneumothorax, haemopericardium, tamponade, pulmonary embolism, and death. Mortality has been reported to range from 0.04% [1] up to 0.28% [3] while the risk of complications increased with the number of leads removed, less physician experience, and female sex [1]. In our case series, no major complications occurred. Male gender predominance among study participants may account at least in part for the absence of any major complications as female gender has been traditionally associated with increased complication rates.

After lead removal, a formal assessment should be contacted to assess whether the patient has still an indication for device-based therapy. Provided the patient meets the criteria to have a new CIED in situ, then it can be done ad hoc, that is, during the same session if a non-infection-related indication had mandated the extraction in the first place [4]. In case of pocket infection with no definite involvement of the intravascular part of the lead, a new system can be implanted in the contralateral site provided that blood cultures are negative 24 h after the procedure and remain negative for at least 72 h afterwards. However, in case of definite device-related endocarditis, a period of at least 14 days should elapse with the patient receiving iv antibiotics before a new device implantation is attempted [4]. In our study, ad hoc implantation of a new CIED was reserved for patients with ventricular (*n* = 10) or atrial leads dysfunction (*n* = 1). In patients with pocket infection (*n* = 25) a new device was implanted after resolution of the infection at the contralateral site (range 3–7 days) whereas in patients with definite CIED-related endocarditis (*n* = 2) a new pacing system was inserted no sooner than 4 weeks as per our institution's protocol.

## 5. Conclusions

In summary, percutaneous extraction of transvenous permanent pacemaker/defibrillator leads can be performed safely and effectively, without major complications even when only basic extraction tools are used. However, the potential risks associated with lead extraction should always be weighed against its benefits. It is of utmost importance to treat each case on an individual basis and candidates for lead extraction should be carefully selected, adequately informed, and actively involved in the decision making process.

## Figures and Tables

**Table 1:** Patient and lead characteristics. Data are mean ± SD or absolute figures (%).

Patient characteristics	*n* = 36	Lead characteristics	*n* = 59
Age (years)	60 ± 5	Implant duration (months)	50 ± 23
Gender (male/female)	27/9	Fixation mode (passive/active)	56/3
Type of device		Lead type	
Pacemaker	25 (69.4)	V pacing	20 (33.9)
ICD	8 (22.2)	A pacing	21 (35.6)
CRT-D	3 (8.4)	VDD pacing	5 (8.5)
Indication for lead extraction		V defibrillator	10 (16.9)
Pocket infection	23 (63.9)	Coronary sinus	3 (5.1)
Endocarditis	2 (5.5)	Extraction system	
V lead dysfunction	10 (27.8)	Traction-type	33 (55.9)
A lead dysfunction	1 (2.8)	Countertraction-type	26 (44.1)
